# Diagnosis of obstructive sleep apnoea

**DOI:** 10.7196/AJTCCM.2019.v25i4.037

**Published:** 2019-12-06

**Authors:** Richard Raine

**Affiliations:** Division of Pulmonology, Department of Medicine, University of Cape Town and Groote Schuur Hospital, South Africa

## Editorial

Obstructive sleep apnoea is a common problem with significant morbidity and mortality. The Wisconsin Sleep Cohort suggests that this could be as high as 4% in men and 2% in women.^[1]^ The prevalence in Southern Africa is unknown, although a recent analysis using published studies of prevalence and algorithms matching population body mass index, race, and geographical location has suggested that the South African population aged between 30 and 69 years has 4 765 612 individuals with an apnoea-hypopnoea index of 15 events per hour or greater. This is ~23% of the at-risk population.^[2]^

The condition has protean manifestations and while most clinicians should recognise the characteristic presentation of obesity, snoring and daytime hypersomnolence, there are other phenotypes which may be more difficult to recognise and respond differently to treatment interventions. Symptom subtypes which predict the incidence of cardiovascular outcomes have recently been described using data from the Sleep Heart Health Study.^[3]^ These range from disturbed sleep (12.2%), minimally symptomatic (32.6%) and moderately sleepy (38.5%) to excessively sleepy (16.7%). The significance of these subtypes, apart from the morbidity related to the sleepiness, is that the excessively sleepy subtype has a three-fold risk of heart failure and approximately double the risk of other cardiovascular diseases compared with other subtypes. This description of subtypes may partially explain the results of the SAVE study, which failed to show benefit of continuous positive airway pressure (CPAP) treatment in a large study of patients with incident cardiovascular disease and moderate-to-severe obstructive sleep apnoea, but minimal sleepiness, randomised to CPAP or usual care.^[4]^ This not to say that treatment with CPAP is ineffective as there are further data from the Sleep Heart Health Study showing improved survival, starting between 6 and 7 years after CPAP initiation, in those treated with CPAP compared with usual care;^[5]^ and a large study from Finland showed that use of CPAP with good compliance (more than 6 hours per night) was associated with a marked decrease in cardiovascular disease events.^[6]^

The varying presentations and increasing evidence of benefit with effective treatment makes it important to confirm the diagnosis of obstructive sleep apnoea. The differing clinical features and responses to treatment suggest that as much diagnostic information as possible be obtained and thus that full polysomnography be performed. In this issue of the *AJTCCM*, van der Colff *et al*. ^[7]^ is a useful demonstration of the information that can be obtained from such investigations. As the study of symptom subtypes suggests, obstructive sleep apnoea is not a homogeneous condition and detailed analysis of events in sleep related to position and sleep stage is very important in understanding mechanisms and pathophysiological phenomena contributing to clinical presentations and responses to interventions.

The American Academy of Sleep Medicine (AASM) recommends that clinical tools and questionnaires should not be used for the diagnosis of obstructive sleep apnoea as commonly used instruments such as the Epworth Sleepiness Scale (sensitivity 0.27 - 0.72; specificity 0.50 - 0.76), Berlin questionnaire (sensitivity 0.76; specificity 0.45), or STOP-BANG (sensitivity 0.93; specifity 0.36) are useful for screening but not diagnosis.^[8]^ In-laboratory polysomnography (as demonstrated in this journal) adds a large amount of detail and precision to the diagnosis but is time- and equipment-intensive.

The AASM has classified sleep studies as:

Level 1 – observed in-laboratory polysomnography;
Level 2 – unobserved, in-laboratory polysomnography;
Level 3 – unobserved, ~ 8 channels of data recorded, including at least nasal pressure, chest and abdominal inductance plethysography, and pulse oximetry;
Level 4 – unobserved, pulse oximetry alone. Level 4 studies are generally inadequate and discouraged.

A level 3 study, home respiratory polygraphy (HRP) or home sleep apnoea test is the most appropriate test for confirming the diagnosis of obstructive sleep apnoea in patients in whom the pre-test likelihood of obstructive sleep apnoea is high, although facilities for level 1 testing should be available should the level 3 study be non-diagnostic. There is good evidence that HRP is as good as polysomnography for diagnosis and titration of CPAP and is considerably cheaper (approximately half the cost).^[9]^

In summary, it is important to confirm the diagnosis of suspected obstructive sleep apnoea as this has implications for treatment and prognosis. A level 3 study is the most cost-effective currently available diagnostic tool.
